# Systemic immune activation in hereditary cancer predisposition syndromes: a cross-sectional study

**DOI:** 10.1186/s12916-026-05188-x

**Published:** 2026-08-25

**Authors:** István Kelemen, Klaudia Horti-Oravecz, Anikó Bozsik, Tímea Pócza, János Papp, István Likó, Patrícia Neuperger, Fanni Balogh, Ágnes Kemény, Petra Nagy, Viktória Vereczki, Szonja Polett Pósa, Lőrinc Sándor Pongor, Dorottya Kövesdi, Henriett Butz, Attila Patócs, Gábor János Szebeni, Vince Kornél Grolmusz

**Affiliations:** 1 Department of Molecular Genetics and the National Tumorbiology Laboratory, National Institute of Oncology, Budapest, Hungary; 2 Lendület “Momentum” Hereditary Cancers Systems Biology Research Group, Hungarian Academy of Sciences - National Institute of Oncology, Budapest, Hungary; 3 Doctoral College, Semmelweis University, Budapest, Hungary; 4 Department of Oncology Biobank, National Institute of Oncology, Budapest, Hungary; 5 HUN-REN-NIO-TTK-HCEMM Oncogenomics Research Group, Budapest, Hungary; 6 Laboratory of Functional Genomics, HUN-REN Biological Research Centre, Core Facility, Szeged, Hungary; 7 Department of Rheumatology and Immunology, Albert Szent-Gyorgyi Medical School and Health Center, University of Szeged, Szeged, Hungary; 8 Department of Physiology and Biochemistry, University of Veterinary Medicine, Budapest, Hungary; 9 Cancer Genomics and Epigenetics Core Group, Hungarian Center of Excellence for Molecular Medicine, Szeged, Hungary; 10 Department of Immunology, Eötvös Loránd University, Budapest, Hungary; 11 Department of Laboratory Medicine, Semmelweis University, Budapest, Hungary; 12 Department of Internal Medicine, Hematology Centre, Faculty of Medicine, University of Szeged, Szeged, Hungary

**Keywords:** Hereditary breast and ovarian cancer syndrome, BRCA1, Lynch syndrome, Pre-cancer immunity, Peripheral immune phenotype, Cancer immunosurveillance, Immune-checkpoint inhibitors, Mass cytometry, Single-cell transcriptomics, Multiparamteric cytokine profiling

## Abstract

**Background:**

Immune surveillance mechanisms contribute to the elimination of precancerous lesions in hereditary cancer predisposition syndromes (HCPSs).

**Methods:**

By combining single-cell transcriptomics, multiparametric mass cytometry and cytokine profiling of the systemic immune environment in 391 individuals among whom 227 are living with HCPSs we investigated phenotypic alterations in cancer-free individuals with HCPS.

**Results:**

A decrease in peripheral B cell abundance and their more differentiated phenotype have been confirmed both in breast cancer patients with germline pathogenic variants in *BRCA1* (gpath(*BRCA1*)) and in patients living with Lynch syndrome (LS). Pre-cancer women with gpath(*BRCA1*) exhibited an activated phenotype of multiple immune cell lineages, similar to those with manifest disease. In LS, B cell phenotypes exhibited the largest changes in response to cancer eradication, while increased peripheral IL-6 levels was detected even in presymptomatic individuals with LS.

**Conclusions:**

HCPS-specific differences in the phenotype of the systemic immune system might be leveraged in future risk-reducing strategies.

**Supplementary Information:**

The online version contains supplementary material available at https://doi.org/10.1186/s12916-026-05188-x.

## Background

Cancer immunosurveillance is a critical process to eliminate preneoplastic lesions and sculpt antitumor immunity which can be leveraged by effective anticancer immunotherapies [1]. In particular, immune-checkpoint inhibitors (ICIs) have revolutionized the therapeutic landscape of cancers with high neoantigen load, providing opportunities for organ-preserving cancer eradication with less adverse effects compared to chemotherapeutic regimens [1–3].

Hereditary cancer predisposition syndromes (HCPSs) are characterized by variable penetrance where large personal differences are observed in cancer risks even in individuals living with the same germline pathogenic variants (gpaths) [4]. Although efforts are being made to include polygenic risk scores into personalized risk stratifications [5], our understanding of how the systemic immune environment modifies cancer risk is scarce. Hereditary breast and ovarian cancer syndrome (HBOC) and Lynch syndrome (LS) are the most frequent HCPSs with potentially life-threatening consequences. Lifetime cancer risks in women living with gpath(*BRCA1*) exceed 70% [6], while gene-specific (*MLH1*, *MSH2*, *MSH6*, *PMS2*) cancer risks in individuals with LS vary between 10 and 50% [7]. Based on groundbreaking results from the past years, facilitation of intrinsic antitumor immunity with ICIs has become a first-line treatment option in the most frequent manifestations in gpath((*BRCA1*)-related HBOC (triple-negative breast cancer, TNBC) and LS (mismatch repair-deficient colorectal cancer) [2, 3, 8].

Presymptomatic individuals living with HBOC and LS exhibit precancer niches. In healthy women living with gpath(*BRCA1*), studies confirmed that luminal progenitor cells are cells-of-origin in breast carcinogenesis [9, 10]. In LS, pathological studies confirmed numerous mismatch repair-deficient (dMMR) foci within healthy colorectal and endometrial tissues [11, 12]. These dMMR foci are probably presenting the so-called shared neoantigens, characteristic of dMMR cancers, against which elevated neoantigen-specific immune responses are already present in presymptomatic individuals with LS [13]. Moreover, clinical trials are investigating if prospective cancer risks can be decreased by stimulating this neoantigen-specific immunity by vaccination [14, 15]. Although lifetime cancer risks are extremely high and preneoplastic lesions are already present in individuals living with HCPSs, studies aiming to characterize the pre-cancer systemic immune environment are lacking. Our previous pilot study was the first to find activation within the systemic immunophenotype in healthy women living with gpath(*BRCA1*) [16] which prompted us to conduct a more comprehensive characterization.

**Fig. 1:**
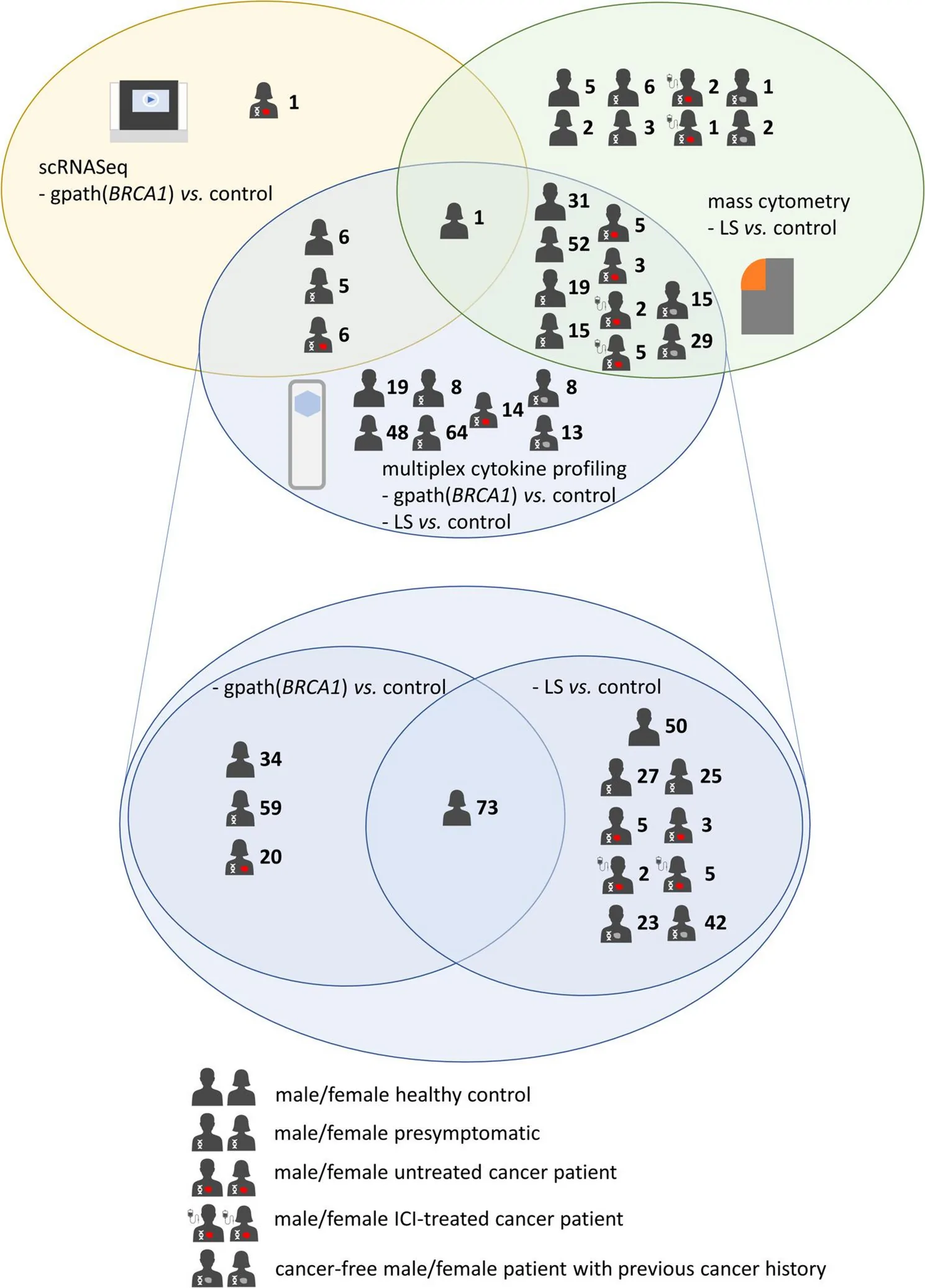
Graphical representation of systemic immunophenotyping methods applied in a cohort of 227 individuals living with HCPS and 164 healthy controls. Individuals with HCPS are differentiated from healthy controls by a double strand symbol. Red markers correspond to untreated cancer, while light grey markers correspond to cancer-free survivors. Overlaps between the cohorts subjected to multiplex cytokine measurements are compared on the lower panel. Abbreviations: gpath germline pathogenic variant, HCPS hereditary cancer predisposition syndrome, ICI immune-checkpoint inhibitor, LS Lynch syndrome, scRNASeq single-cell RNA sequencing

Here, we present results from a comprehensive immunoprofiling approach including single-cell transcriptomics, mass cytometry and multiplex cytokine profiling to characterize the systemic immune phenotypes of 227 individuals living with HCPS as compared to 164 healthy controls living without HCPSs (Fig. 1). We sought to determine whether systemic immune alterations are present prior to cancer occurrence and how these change with cancer development and eradication.

## Methods

### Patient accrual and genetic diagnosis

Cancer patients and healthy individuals referred to genetic counseling at the Department of Molecular Genetics, National Institute of Oncology were included in this study between March 24, 2021, and March 31, 2025. Following genetic counseling, patients provided informed consent to be included in the study. As described earlier [16], only individuals without concurrent (respiratory, gastrointestinal or genitourinary) infections or fever and without previous diagnosis of autoimmune diseases were included, while individuals undergoing immunomodulatory therapies (e.g., local or systemic corticosteroid treatment) were excluded. All participants were adult White people and individuals with HCPSs were analyzed in comparison with age- and sex-matched healthy control cohorts. Baseline characteristics of study subjects are detailed in Additonal File 1: Supplementary Tables S1-S10.

Following genetic counseling and written informed consent, DNA was isolated from peripheral blood using the Gentra Puregene Blood Kit (Qiagen, Hilden, Germany) according to the manufacturer’s instructions. For individuals diagnosed with cancer, germline mutation analysis of *BRCA1*, *MLH1*, *MSH2*, *MSH6*, *PMS2*, and *EPCAM* genes was conducted using next-generation sequencing. Libraries were prepared with TruSight Hereditary Cancer Panel (Illumina Inc., San Diego, CA, USA), and sequencing was performed either on a MiSeq or a NextSeq 550Dx instrument (Illumina Inc., San Diego, CA, USA). Bioinformatic analyses were carried out in DRAGEN 4.2.1 environment (Illumina Inc., San Diego, CA, USA). Healthy individuals underwent cascade testing for familial pathogenic variants. PCR-amplified amplicons were analyzed using BigDye Terminator Kit (Thermo Fisher Scientific, Waltham, MA, USA) and subjected to gold-standard Sanger dideoxy sequencing. Variants were detected *via* capillary electrophoresis on a 3500 Genetic Analyzer (Thermo Fisher Scientific). Interpretation followed ACMG/AMP guidelines [17]. Cancer patients were included only if they had not yet received treatment for their current malignancy (treatment-naive status) or were currently undergoing immune-checkpoint inhibitor treatment with pembrolizumab (ICI-treated LS cohort). Cancer-free LS individuals were included following eradication of the disease (cancer-free) and, if radio- or systemic antineoplastic therapies were applied, at least 3 years had passed since their administration. Presymptomatic healthy individuals (gpath(*BRCA1*) healthy cohort and presymptomatics LS cohort) carry a germline genetic predisposition to cancer but have not yet been diagnosed with cancer. All participants were informed about the study design and procedures, and written informed consent was obtained before sample collection. The study was approved by the Scientific and Research Ethics Committee of the Medical Research Council of Hungary (approval number: NNK 53720-7/2019/EÜIG).

### PBMC and plasma isolation

PBMC and plasma samples were isolated within 4 h of venipuncture using the Pancoll reagent (PanBiotech GmbH, Cat. No.: P04-601000) as earlier reported [16, 18]. PBMC samples were cryopreserved in liquid nitrogen, while plasma samples were stored at -80 °C until further processing.

### Single-cell RNA sequencing

PBMC samples were rapidly thawed in a 37 °C water bath for 2 min, washed with 5 ml PBS solution (Corning Inc., Cat. No.: 21-040-CV) containing 10% FBS (PanBiotech GmbH, Cat. No.: P30-19375) and centrifuged at 300 g for 5 min at room temperature (RT). Cells were counted using a standard counting chamber and the cell suspension was diluted to 1000 cells/µl. Single-cell libraries were generated using the Chromium Next GEM Single Cell 3ʹ library preparation kits (v3.1, Cat. No.s: PN-1000268, PN-1000127, PN-1000215) using a Chromium Controller instrument (all from 10X Genomics Inc.) following the manufacturer’s instructions. Sequencing was performed on a NovaSeq 6000 instrument (Illumina Inc.) using an S4 flow cell.

Gene expression – cell matrices were created with CellRanger (v.7.1.0) using the refdata-gex-GRCh38-2020-A transcriptome version, were deposited in Zenodo and were further processed in Seurat (version 5.1.0). Data from 19 individual samples (7 healthy women without hereditary cancer predisposition, 5 healthy women with gpath(*BRCA1*) and 7 women with untreated triple-negative breast cancer with gpath(*BRCA1*)) were merged. In total, data from 100,132 cells remained after quality control step (filtering out cells with nFeatureRNA < 1250 and nFeatureRNA > 5000 and cells with larger than 8% of mitochondrial gene expression) which were normalized and transformed using SCTransform and integrated using Harmony. Clustering of the cells was performed using FindNeighbors and FindClusters and was represented using UMAP. Cluster annotations of immune cell subpopulations were performed using marker genes and were cross-referenced using Celltypist [19]. Within the five most abundant peripheral immune cell types (B cells, CD4^+^ T cell, CD8^+^ T cells, NK cells, monocytes), subclustering was also performed using FindNeighbors and FindClusters to investigate phenotypically different subpopulations. Differential gene expression was performed using FindMarkers. CellChat was used to identify differently affected secreted signaling pathways between B cell subcluster B_1 and CD4^+^ T cell subcluster CD4_T_8 [20]. Cell-specific pathway activity scores were calculated using AUCell package [21] and were restricted to immune-related pathways included in the ImmuneSigDB subset of C7: immunologic signature gene sets in the Human Molecular Signatures Database (https://www.gsea-msigdb.org/gsea/msigdb/human/collections.jsp). Cell differentiation scores within the 5 abundant peripheral immune cell compartments were calculated using the pseudotime analysis within the Monocle3 package [22] with most naive cell subclusters defined as the origin of ordering.

### Mass cytometry

Samples were processed for mass cytometry experiments as previously reported [16].

Cryovials containing PBMC suspensions were thawed in a 37 °C water bath for 2 min and transferred into 9 mL of complete RPMI-1640 medium (cRPMI) containing 100 U/mL penicillin (Merck, USA), 100 µg/mL streptomycin (Merck, USA), 2 mM L-glutamine (Capricorn Scientific GmbH, diluted from 200 mM stock), and 10% FBS (Euroclone, Italy). Cells were centrifuged at 370 × g for 6 min at RT, washed once in 10 mL cRPMI, and counted using a standard cell counting chamber. Viability was assessed by trypan blue staining (Thermo Fisher Scientific, Waltham, MA, USA). For each sample, 3 × 10*6 PBMCs were transferred into a 96-well U-bottom, cell-repellent plate (Greiner Bio-One Ltd, UK) in 200 µL cRPMI and incubated for 2 h at 37 °C in a 5% CO₂ incubator. Thereafter, cells were collected and washed twice with Maxpar Cell Staining Buffer (MCSB; Standard Biotools, San Francisco, CA, USA).

Cells were resuspended in 200 µL MCSB and transferred into 1.5 mL centrifuge tubes. After adding 800 µL MCSB, cells were washed at 370 × g for 6 min at RT. They were then resuspended in 47.5 µL MCSB containing 2.5 µL TruStain FcX (1:20 V/V%; BioLegend, San Diego, CA, USA) and incubated for 10 min at RT. PBMC samples from 8 patients were individually barcoded using CD45 antibodies (clone HI30; Standard Biotools, San Francisco, CA, USA) conjugated to distinct metal isotopes (⁸⁹Y, ¹⁰⁶Cd, ¹¹⁰Cd, ¹¹¹Cd, ¹¹²Cd, ¹¹³Cd, ¹¹⁴Cd, ¹¹⁶Cd) at a final concentration of 1:100 per antibody. This method has been described previously [16]. Cells were incubated at 4 °C for 30 min, washed twice in 1 mL MCSB at 370 × g for 6 min at RT, resuspended in 1 mL MCSB, and counted. From each individual, 5 × 10^5^ cells were pooled into 5 mL centrifuge tubes and centrifuged at 370 × g for 6 min at RT. Cells were resuspended in 275 µL MCSB and transferred into lyophilized antibody-containing tubes from the Maxpar Direct Immune Profiling Assay (Standard Biotools, San Francisco, CA, USA). After 30 min of incubation at RT, cells were washed twice in 2 mL MCSB, centrifuged at 370 × g for 6 min at RT, and fixed in 1.6% formaldehyde (Pierce, Thermo Fisher Scientific, Waltham, MA, USA) for 10 min at RT. Fixed and labeled cells were centrifuged at 800 × g for 6 min at RT, resuspended in 800 µL Fix & Perm solution containing ^191^Ir-^193^Ir DNA intercalator (1:1000 V/V%; Standard Biotools, San Francisco, CA, USA), and incubated overnight.

Pooled cells were washed three times in 4 mL MCSB at 800 × g for 6 min at RT, then filtered through a 30 μm CellTrics gravity filter (Sysmex, Görlitz, Germany). Cell concentrations were adjusted to 8 × 10^5^/mL in Cell Acquisition Solution (CAS; Standard Biotools, San Francisco, CA, USA). EQ four-element calibration beads (Standard Biotools, San Francisco, CA, USA) were added at a 1:10 ratio (V/V%). Samples were measured on a calibrated Helios mass cytometer equipped with a WB injector (Standard Biotools, San Francisco, CA, USA). For each pool, 2.5 × 10^6^ events were recorded to ensure at least 1 × 10^5^ intact cells per individual sample, enabling detection of rare cell populations. Flow cytometry standard (FCS) files generated during acquisition were randomized and normalized using CyTOF Software (Standard Biotools, San Francisco, CA, USA; version 7.0.8493) with default FCS processing settings.

Randomized and normalized FCS files were uploaded to the Cytobank Premium platform (Beckman Coulter). After the exclusion of normalization beads, dead cells, debris and doublets, manual debarcoding was performed. FCS files containing CD45^+^ live cells were exported, and deposited in Zenodo, with further evaluation carried out in R (v4.4.2) environment. The compensation method, clustering and dimensionality reduction performed with ConsensusClusterPlus and FlowSOM algorithms were adapted from Crowell et al. [23]. The fcs files were integrated, compensated and transformed with CATALYST and flowCore R packages. Following signal spillover compensation, CyTOF marker intensities underwent inverse hyperbolic sine transformation with a cofactor of 5. For the identification of major cell populations, clustering based on the self-organizing-map method (FlowSOM) was performed on the compensated and transformed files. 10 major immune populations were distinguished based on the relative median marker expression levels of CD3, CD4, CD8a, CD11c, CD14, CD19, CD20, CD56, TCRgd and HLA-DR type markers. From a representative sampling of 19.9 million cells and using relative median marker expression values of 29 markers, t-SNE (t-distributed stochastic neighbor embedding) and UMAP (Uniform Manifold Approximation and Projection) based visualizations of the human peripheral immune system were generated. Subclustering of the identified major immune populations into subpopulations was performed using ConsensusClusterPlus, FlowSOM and UMAP algorithms.

### Enzyme-linked immunosorbent assay analyses

#### Macrophage inhibitory factor assay

Centrifuge tubes containing plasma were thawed at room temperature within 10 min, followed by centrifugation at 10,000 g for 10 min at 5 °C. The resultant supernatant was diluted 20-fold. 25 µL of plasma sample was added to 475 µL of Calibrator Diluent RD5-20 (R&D Systems, Minneapolis, MN, USA).

Quantitative determination of macrophage migration inhibitory factor (MIF) concentration in plasma was performed using the Quantikine™ ELISA Human MIF Immunoassay Kit (R&D Systems, Minneapolis, MN, USA). Preparation of reagents (Wash Buffer, Substrate Solution, Calibrator Diluent RD5-20, Human MIF Standard) and assembly of the assay were carried out according to the manufacturer’s instructions.

#### C-C Motif Chemokine Ligand 19 and 21 assays

Centrifuge tubes containing plasma samples were thawed at room temperature within 10 min, followed by centrifugation at 2000 g for 10 min at 5 °C. The resultant supernatant was diluted 4-fold: 25 µL of plasma sample was added to 75 µL of Sample Diluent 50 (Abcam, Cambridge, Cambridgeshire, UK).

Quantitative determination of C-C Motif Chemokine Ligand 19 and 21 (CCL19 and CCL21) concentration in plasma was performed using the Human CCL19 SimpleStep ELISA Kit and the Exodus 2 (CCL21) Human SimpleStep ELISA Kit (Cat. No.: ab245723 and ab193759, respectively, both from Abcam, Cambridge, Cambridgeshire, UK). Preparation of reagents and assembly of the assay were carried out according to the manufacturer’s instructions.

Assembled 96-well microplates were measured within 30 min after assay completion using an Agilent BioTek Epoch 2 Microplate Spectrophotometer (Thermo Fisher Scientific, Waltham, MA, USA). The microplate reader was set to 450 nm with correction wavelengths of 540 and 570 nm. Results of standards, controls, and sample duplicates were averaged, and the mean optical density (O.D.) of the zero standard was subtracted from the measured values. A standard curve was generated by plotting standard concentrations on the Y-axis against sample concentrations on the X-axis, and a best-fit linear curve was drawn through the points on a log/log graph.

### Multiparametric cytokine profiling

The measurement of plasma proteins was performed as described previously by our group with minor modifications [24, 25]. Briefly, blood samples were collected in EDTA tubes, and the plasma fraction obtained after centrifugation was stored at -80 °C before running the assay. Luminex xMAP (MAGPIX^®^) technology was used to determine the protein concentrations of 25 distinct cytokines (Eotaxin/CCL11, GM-CSF, IFNa, IFNg, IL-1β, IL-10, IL-12 (p40/p70), IL-13, IL-15, IL-17 A, IL-1RA, IL-2, IL-2R, IL-4, IL-5, IL-6, IL-7, IL-8/CXCL8, IP-10/CXCL10, MCP-1/CCL2, MIG/CXCL9, MIP-1a/CCL3, MIP-1b/CCL4, RANTES/CCL5, TNFa) performing the Human Cytokine Magnetic 25-Plex Panel (Invitrogen, Cat. No.: LHC0009M) according to the instructions of the manufacturer. Briefly, all samples were thawed, centrifuged at 12,000 g for 10 min to clarify the samples and tested blindly. 25 µl of capture antibody-coated, fluorescent-coded beads were added to the wells, and after two washing steps, 50 µl of Incubation Buffer was added to each well, 100 µl of standard/background to standard wells, 50 µl of Assay Diluent and 50 µl of undiluted plasma samples to the sample wells and it was incubated at 4 °C overnight. Following 2 washing steps, biotinylated detection antibody mixture was added to the plate and incubated on a plate shaker at RT for 1 h. After 2 washing steps, streptavidin-RPE was added to the plate and incubated on a plate shaker at RT for 30 min. After 3 washing steps, 150 µl Wash Solution was added to each well and the plate was incubated on a plate shaker for an additional 5 min and read on the Luminex MAGPIX^®^ instrument. Luminex xPonent 4.2 software was used for data acquisition. Five-PL regression curves were generated to plot the standard curves for all analytes by the Belysa (Merck) software calculating with bead median fluorescence intensity values. Concentration values of the plasma were expressed as pg/ml. Cytokines which were measurable in at least 80% of the samples were further analyzed (CCL11, IL-1b, IL-1RA, IL-2, IL-2R, IL-6, IL-12, IP-10, MCP-1, MIG, MIP-1a, MIP-1b, TNFa).

### Statistical analysis

Analysis of relative distributions and median marker intensity values of the major and subpopulations was performed in R (v4.4.2) environment and in Graphpad Prism (v.8.0.1). For the study groups, normality testing (Shapiro-Wilk test) and variance testing (Levene test) were conducted. For group comparisons, in case of normal distribution and homogeneity of variances, classical ANOVA was applied, while in case of normal distribution but heterogeneous variances, Brown-Forsythe ANOVA was used. In the case of non-normal distribution, Kruskal-Wallis test was applied. P-values obtained from multiple comparison tests were corrected using a two-stage linear step-up procedure of Benjamini, Krieger and Yekutieli with a q value cutoff at 0.1.

To assess the relationship between age, relative distributions and median marker intensity values, Pearson correlation and simple linear regression models were computed separately within each condition. For each model, correlation coefficients (r), correlation p-values, regression slopes, regression p-values and coefficients of determination (R^2^) were obtained. Contingency tables were subjected to chi^2^ test to investigate if distributions in sex, affected gene or manifest cancer type differed between LS cohorts. Differences between groups were considered significant at p-value of *p* < 0.05*; *p* < 0.01**; *p* < 0.001***.

## Results

### Single-cell transcriptomics reveal a universally activated phenotype in different immune cell lineages in women with gpath(*BRCA1*)

**Fig. 2:**
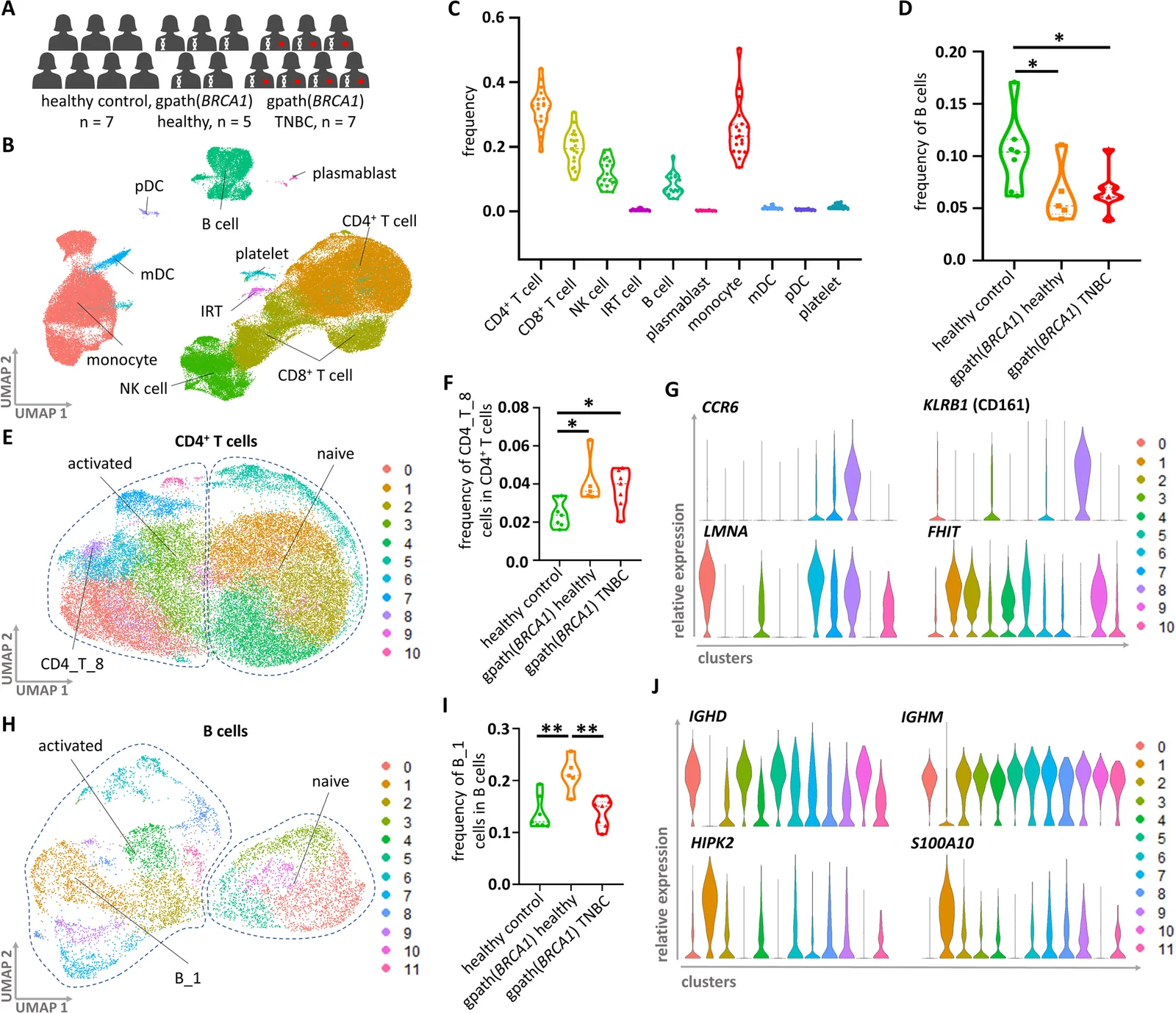
Annotation and relative abundance analysis of peripheral immune cell populations based on single-cell transcriptomics in gpath(*BRCA1*) carrier women. Graphical representation of study participants, extended data can be found in Additional File 1: Supplementary Table S1 and Additional File 2: Supplementary Figure S1 (**A**). UMAP representation of peripheral immune cells clustered into 10 main cell types (**B**). Relative abundance of each cell type in each sample (**C**). Relative abundance of B cells within PBMC in various study groups (**D**). UMAP representation of CD4^+^ T cell subclusters, extended data can be found in Additional File 2: Supplementary Figure S2 (**E**). Cells are color-coded by their respective CD4^+^ T cell subclusters. Relative abundance of CD4_T_8 subcluster within CD4^+^ T cells in various study groups (**F**). Expression of selected CD4_T_8-specific subcluster marker genes in CD4^+^ T cell subclusters, extended data can be found in Additional File 1: Supplementary Table S2 (**G**). Violin plots are color-coded based on their respective CD4^+^ T cell subcluster. UMAP representation of B cell subclusters, extended data can be found in Additional File 2: Supplementary Figure S2 (**H**). Cells are color-coded by their respective B cell subclusters. Relative abundance of B_1 subcluster within B cells in various study groups (**I**). Expression of selected B_1-specific subcluster marker genes in B cell subclusters, extended data can be found in Additional File 1: Supplementary Table S3 (**J**). Violin plots are color-coded based on their respective B cell subcluster. Asterisks correspond to statistical significance: *p* < 0.05*; <0.01**. On violin plots, dashed lines correspond to median values, while dotted lines border interquartile ranges. Abbreviations: gpath germline pathogenic variant, IRT interferon-responsive T, NK natural killer, mDC myeloid dendritic cell, pDC plasmacytoid dendritic cell

Single-cell transcriptomic analysis of 100,132 peripheral mononuclear cells originating from 19 women (7 healthy women without germline cancer predisposition, 5 healthy women living with gpath(*BRCA1*) and 7 treatment-naive TNBC patients (Additional File 1: Supplementary Table S1, Additional File 2: Supplementary Fig. 1) living with gpath(*BRCA1*)) revealed 10 primary immune cell clusters, frequencies of which mirrored the basic distribution of peripheral immune cell lineages (Fig. 2A-C). The relative abundance of B cells was decreased in healthy and cancer-bearing gpath(*BRCA1*) carrier women compared to healthy controls (healthy control: 0.103 ± 0.036 vs. gpath(*BRCA1*) healthy: 0.064 ± 0.028, *p* = 0.034; vs. gpath(*BRCA1*) TNBC: 0.067 ± 0.021, *p* = 0.033), Fig. 2D). Subclustering was performed on the five most abundant immune populations (CD4^+^ T cells, CD8^+^ T cells, B cells, NK cells and monocytes) which revealed one CD4^+^ T cell (CD4_T_8 subcluster, Fig. 2E-F, Additional File 2: Supplementary Figure S2A) and one B cell (B_1 subcluster, Fig. 2H-I, Additional File 2: Supplementary Figure S2B) subclusters exhibiting increased relative frequencies in healthy gpath(*BRCA1*) carrier women (CD4_T_8 subcluster relative frequencies: healthy control: 0.024 ± 0.007 vs. gpath(*BRCA1*) healthy: 0.041 ± 0.013, *p* = 0.017; vs. gpath(*BRCA1*) TNBC: 0.038 ± 0.010, *p* = 0.012, B_1 subcluster relative frequencies: gpath(*BRCA1*) healthy: 0.212 ± 0.033 vs. healthy control: 0.137 ± 0.032, *p* = 0.007; vs. gpath(*BRCA1*) TNBC: 0.138 ± 0.027, *p* = 0.0095). Both subclusters are characterized by activated phenotypes. In the case of CD4_T_8 subcluster (Fig. 2G, Additional File 1: Supplementary Table S2), the decreased expression of *FHIT* and increased *LMNA* expression confirmed the activated phenotype [26, 27], while the high expression of key marker genes *CCR6* and *KLRB1* confirms Th17-polarization [28, 29]. Low *IGHD* and *IGHM* expression accompanied by high *HIPK2* and *S100A10* expressions in the B_1 subcluster is consistent with a class-switched, inflammation-adapted B cell subpopulation (Fig. 2J, Additional File 1: Supplementary Table S3) [30].

**Fig. 3:**
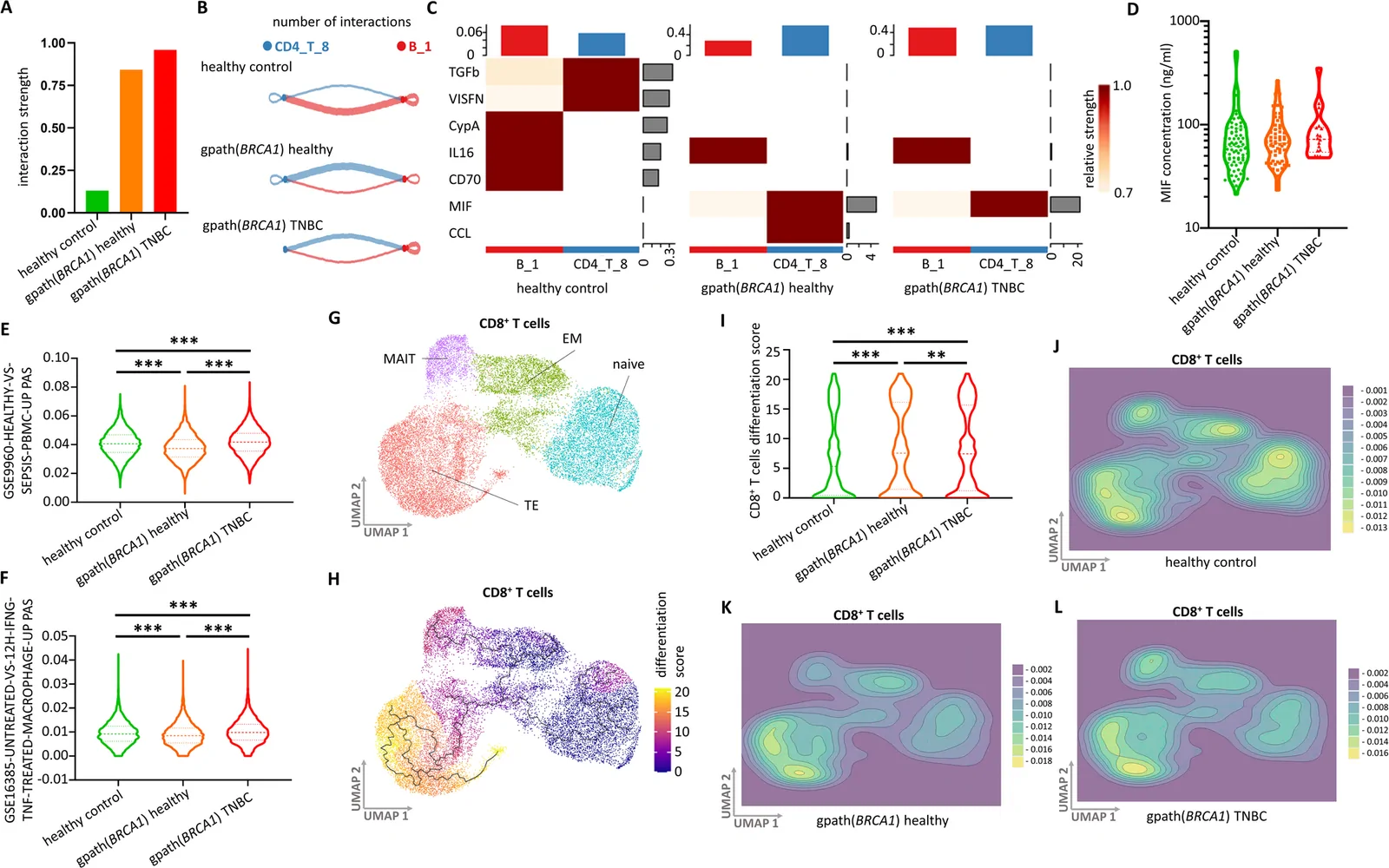
Detailed phenotypic analyses of single-cell transcriptomics data from PBMC samples from women with gpath(*BRCA1*). Graphical representation of interaction strengths between CD4_T_8 and B_1 cells in different study groups as characterized by CellChat (**A**). Graphical representation of the number of interactions between CD4_T_8 and B_1 cells in different study groups as characterized by CellChat (**B**). Note: Number of interactions from CD4_T_8 to B_1 cells are highlighted in blue, while signals from B_1 cells to CD4_T_8 cells are colored red. Relative role of specific pathways among total interactions between CD4_T_8 and B_1 cells as characterized by CellChat (**C**). Significant pathways affected in at least one study group are compared. The heatmap demonstrates the relative strengths of each cytokine pathway in the three study groups in both the B_1 and the CD4_T_8 subcluster. The right panels provide inter-pathway while the upper panels provide inter-cell type comparisons in each study group. Peripheral plasma MIF levels in a larger cohort of gpath(*BRCA1*) carriers and healthy controls (**D**). Extended data including baseline characteristics of this cohort can be found in Additional File 1: Supplementary Table S4 and Additional File 2: Supplementary Figure S3. Pathway activity scores of monocytes between study groups regarding pathways GSE9960-HEALTHY-VS-SEPSIS-PBMC-UP (**E**) and GSE16385-UNTREATED-VS-12 H-IFNG-TNF-TREATED-MACROPHAGE-UP (**F**). Note: lower values reflect higher activation in disease/treated groups compared to control cohorts. Extended data for further immune-related pathways in monocytes can be found in Additional File 1: Supplementary Table S6. UMAP representation of CD8^+^ T cell subsets (**G**). UMAP representation of CD8^+^ T cells color-coded by differentiation scores (**H**). Comparison of CD8^+^ T cell differentiation scores between various study groups (**I**). Extended data for further main immune cell types can be found in Additional File 2: Supplementary Figure S4. Cell density plots on UMAP representation of peripheral CD8^+^ T cells in healthy control (**J**), gpath(*BRCA1*) healthy (**K**) and gpath(*BRCA1*) TNBC (**L**) samples color-coded by relative densities. Asterisks correspond to statistical significance: *p* < 0.01**; <0.001***. On violin plots, dashed lines correspond to median values, while dotted lines border interquartile ranges. Abbreviations: CCL chemokine ligand pathway, CD70 cluster of differentiation 70, CypA cyclophilin A pathway, EM effector memory CD8^+^ T cells, IL16 interleukin 16 pathway, MAIT mucosal-associated invariant T cells, MIF macrophage migration inhibitory factor, PAS pathway activity score, TE terminal effector CD8^+^ T cells, TGFb transforming growth factor beta-pathway, VISFN visfatin-pathway

As these results confirmed our earlier observations [16] of increased activated CD4^+^ T and B cell subpopulations in healthy and TNBC-bearing gpath(*BRCA1*) carriers in an independent cohort, we aimed to investigate their functional relationship. In particular, we investigated the interaction strengths between these two subclusters based on the transcriptional levels of key members of secreted signaling pathways using CellChat. A striking increase in interaction strengths in healthy gpath(*BRCA1*) and gpath(*BRCA1*) TNBC was observed as compared to healthy controls (Fig. 3A). Additionally, communication directions between these two cell types, as inferred by CellChat, changed in women with gpath(*BRCA1*), with the number of CD4_T_8-to-B_1 cell interactions increasing in both gpath(*BRCA1*) study groups (Fig. 3B). Detailed analysis of specific signaling pathways revealed that the inflammatory cytokine, macrophage migration inhibitory factor (MIF), might primarily mediate the strengthened interactions in gpath(*BRCA1*) groups (Fig. 3C). Since altered interactions between smaller subsets of CD4^+^ T and B cells might not necessarily translate to altered concentrations in peripheral plasma, we performed plasma-based cytokine analysis on a larger cohort (Additional File 1: Supplementary Table S4, Additional File 2: Supplementary Figure S3). Measurement of MIF levels in plasma samples from 77 gpath(*BRCA1*) carriers (among which 59 were healthy, presymptomatic and 18 were treatment-naive TNBC patients) and 79 healthy women without HCPS revealed no significant differences between study groups; however, a slight increase in plasma MIF levels in patients with TNBC can be observed (*p* = 0.320, Fig. 3D).

Differential gene expression analyses focusing on the five main immune cell populations revealed significantly differently expressed genes between study groups (Additional File 1: Supplementary Table S5). Overall, 3514 genes were found to be differently expressed in monocytes, followed by 2824 genes in CD4^+^ T cells, 1799 genes in NK cells, 1256 genes in CD8^+^ T cells and 905 genes in B cells. The AUCell algorithm was used to compare pathway activity scores of immune-related pathways between groups. Altogether, 106 pathway alterations were detected in monocytes, followed by 76 in NK cells, 37 in CD4^+^ T cells, 26 in CD8^+^ T cells and 24 in B cells (Additional File 1: Supplementary Table S6). Within monocytes, multiple independent pathway analyses signaled a slightly more inflammatory phenotype in healthy gpath(*BRCA1*) carriers (as demonstrated by the decrease of pathway activity scores related to normal vs. sepsis (healthy control: 0.041 ± 0.009, gpath(*BRCA1*) healthy: 0.038 ± 0.009, *p* < 0.0001) and untreated vs. TNF-alpha-and-IFN-gamma-treated comparisons (healthy control: 0.010 ± 0.005, gpath(*BRCA1*) healthy: 0.009 ± 0.005, *p* < 0.0001), Figs. 3E-F). Since these differences appear to be modest, further validation in independent cohorts are needed to confirm biological reproducibility. Nevertheless, as these results all signaled a more activated phenotype in multiple peripheral immune lineages, we also investigated the distribution of differentiation scores of each main immune cell type. Differentiation scores were calculated using the pseudotime analysis within Monocle 3 and compared between study groups. Mean differentiation scores were elevated in four (CD4^+^ and CD8^+^ T cells, B cells and monocytes) out of five main immune cell lineages in gpath(*BRCA1*) carriers with the most pronounced increase within CD8^+^ T cells (median and interquartile ranges of CD8^+^ T cell differentiation scores: healthy control: 5.33 (0.441–14.2) vs. gpath(*BRCA1*) healthy: 7.55 (1.45–16.2), *p* < 0.0001; vs. gpath(*BRCA1*) TNBC: 7.45 (1.21–15.7), *p* < 0.0001, Fig. 3G-I and Additional File 2: Supplementary Figure S4), resulting from a clear shift towards the activated, effector memory phenotype (Figs. 3J-L).

### Peripheral cytokine profiles of women with gpath(*BRCA1*) do not mirror inflammatory alterations

To investigate if activated cellular phenotypes corresponding to an elevated inflammatory phenotype correlate with the peripheral cytokine profile, parallel profiling of 13 secreted cytokines and chemokines from plasma samples of 79 gpath(*BRCA1*) carriers (59 healthy presymptomatic women and 20 with treatment-naive TNBC) were compared with 107 healthy women living without hereditary cancer predisposition (Additional File 1: Supplementary Table S7 and Additional File 2: Supplementary Figure S5). Among the investigated cytokines, none exhibited significant differences between study groups (Additional File 2: Supplementary Figures S6-S7). This result argues against a universal inflammatory state in healthy gpath(*BRCA1*) carrier women and proposes that tissue-specific alterations could drive the elevated systemic frequency of pro-inflammatory CD4^+^ T and B cells.

### Mass cytometry profiling of peripheral lymphocytes reveals increased frequencies of naive B cell phenotypes during cancer eradication in LS

**Fig. 4:**
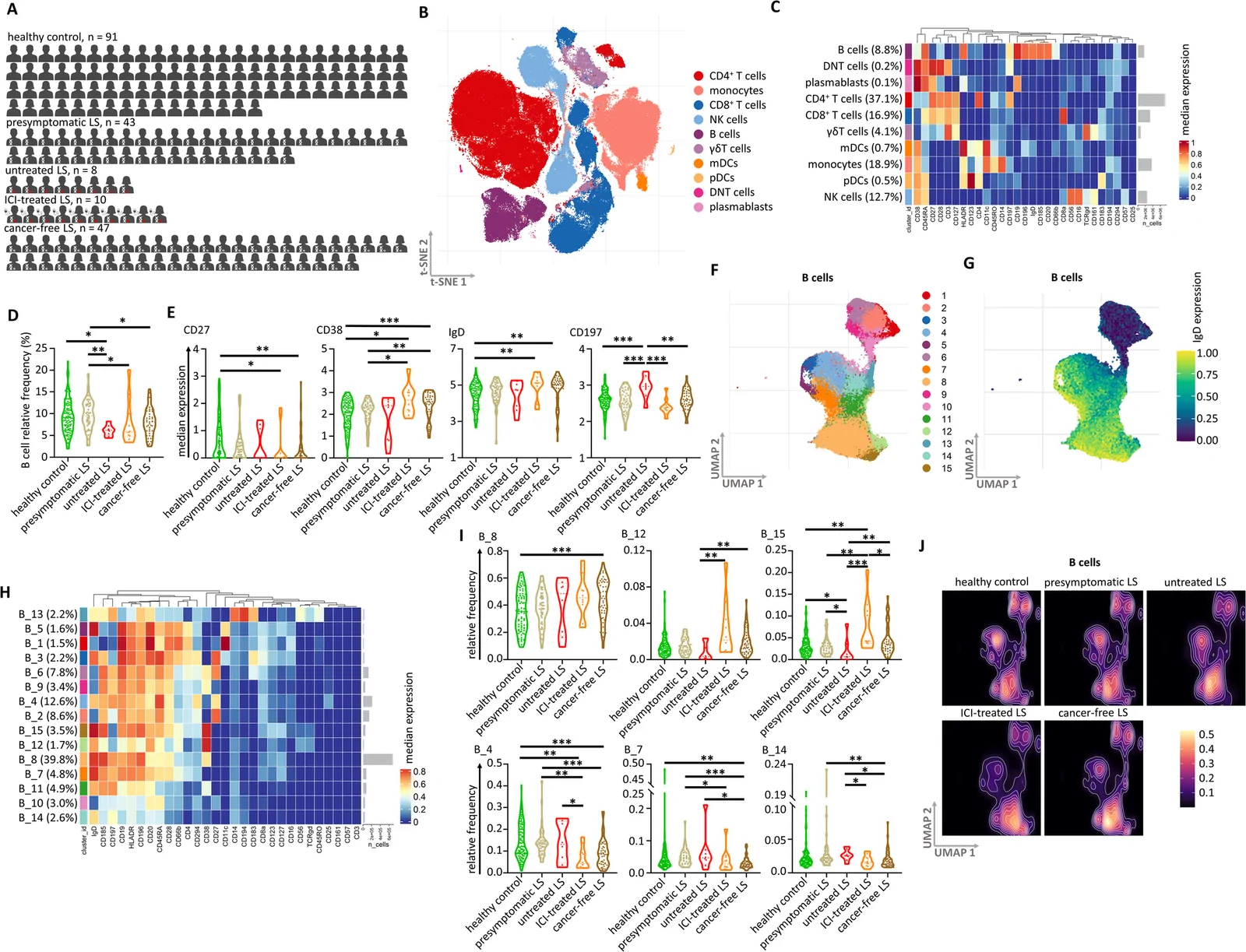
Mass cytometry-based peripheral immunophenotyping focusing on B cell phenotypes in Lynch syndrome. Graphical representation of study participants, extended data can be found in Additional File 1: Supplementary Table S8 and Additional File 2: Supplementary Figure S8 (**A**). t-SNE representation of the detected immune cell populations (**B**). Extended data including UMAP representation and marker-specific expression heatmaps can be found in Additional File 2: Supplementary Figures S9-S10. Heatmap of median marker expressions and relative frequencies of each identified peripheral immune cell populations (**C**). Comparison of peripheral B cell frequencies between study groups (**D**). Extended data including age-related analysis is presented in Additional File 2: Supplementary Figure S11. Median marker expressions of CD27, CD38, IgD and CD197 on B cells in each subject (**E**). Extended data including age-related analysis is presented in Additional File 2: Supplementary Figure S12. UMAP representation of peripheral B cell subclusters (**F**). UMAP representation of peripheral B cells color-coded by IgD expression (**G**). Extended data for further marker expressions can be found in Additional File 2: Supplementary Figure S13. Heatmap of median marker expressions and relative frequencies of each identified peripheral B cell subclusters (**H**). Comparison of relative frequencies of B cell subclusters between study groups (**I**). Extended data including age-related analyses are presented in Additional File 2: Supplementary Figure S14. Cell density plots on UMAP representation of peripheral B cells in different study groups color-coded by relative densities (**J**). On violin plots, dashed lines correspond to median values, while dotted lines border interquartile ranges. Asterisks correspond to statistical significance: *p* < 0.05*; < 0.01**; < 0.001***. Abbreviations: DNT cells double negative T cells, NK natural killer, mDCs myeloid dendritic cells, pDCs plasmacytoid dendritic cells

To investigate the systemic immune phenotype of Lynch syndrome, we conducted a large-scale mass cytometry investigation. 108 individuals with LS were analyzed in comparison with an age-matched healthy control cohort (*n* = 91) without HCPSs (Fig. 4A, Additional File 2: Supplementary Figure S8A, Additional File 1: Supplementary Table S8). The LS cohort was subgrouped based on cancer and treatment status into presymptomatic (negative cancer history), untreated (treatment-naive cancer), ICI-treated (immune-checkpoint inhibitor-treated cancer) and cancer-free (cancer-free survivor patients with cancer history) study groups. The subgrouping of the LS cohort allowed our analyses to reflect the course of events in LS (pre-cancer, cancer occurrence, eradication). As these subgroups exhibited differences in distributions of age but not of sex, affected gene (*MLH1*, *MSH2*, *MSH6*, *PMS2*, *EPCAM*) or cancer type (Additional File 2: Supplementary Fig. 8B, Additional File 1: Supplementary Table S9), further examinations were complemented with correlation analyses for age. Based on expression patterns of the investigated cell surface markers, we identified 10 peripheral immune cell populations (Fig. 4B-C, Additional File 2: Supplementary Figures S9-S10) with highly similar peripheral frequencies as in our earlier study [16]. Among the main immune cell types, a decrease in B cell proportions was found in patients with untreated cancer (untreated LS: 6.13 ± 1.25 vs. healthy control: 9.10 ± 3.82, *p* = 0.013; vs. persymptomatic LS: 9.64 ± 3.38, *p* = 0.003; Fig. 4D), highly similar to what has been observed in gpath(*BRCA1*) carrier women with untreated TNBC (Fig. 2D). Peripheral B cell frequencies did not correlate with the age of subjects (Additional File 2: Supplementary Figure S11). The decrease of CD27 expression and increase in CD38 and IgD expression on B cells in ICI-treated cancer patients and cancer-free survivors indicate a shift towards more naive B cell phenotypes in these cohorts, while the increase of CD197 expression on B cells in untreated LS patients can correlate with an increased migratory phenotype of these cells towards secondary lymphatic tissues (Fig. 4E). Importantly, the expression of these proteins in B cells did not correlate with the age of the subjects (Additional File 2: Supplementary Figure S12).

To dissect how these phenotypic changes are reflected in alterations between B cell subpopulations, subclustering was performed specifically on B cells. 15 different subclusters were identified with IgD expression being one of the main differentiating factors between them (Figs. 4F-H, Additional File 2: Supplementary Figure S13). In particular, the relative frequencies of 6 (B_8, B_12, B_15, B_4, B_7 and B_14) of these subclusters exhibited significant alterations between study groups (Fig. 4I, Additional File 2: Supplementary Figure S14). Changes in these relative abundances can be categorized in two patterns. Subclusters B_8, B_12 and B_15 are more frequent in ICI-treated LS and cancer-free LS groups, with B_12 and B_15 being less frequent in untreated LS cancer patients. These three subclusters are all naive B cells exhibiting high expression of IgD, and in the cases of B_12 and B_15, CD38. High expression of CD38 on IgD^+^ B cells is characteristic to transitional B cells, which are considered the most naive B cells in the periphery [31]. Based on the 4-fold increases of B_12 and B_15 frequencies during ICI therapy (mean B_12 relative frequency in untreated LS: 0.0087 ± 0.01, in ICI-treated LS: 0.033 ± 0.032, *p* = 0.006, mean B_15 relative frequency in untreated LS: 0.018 ± 0.028, in ICI-treated LS: 0.077 ± 0.058, *p* < 0.0001) it is plausible that an increase in bone marrow B cell efflux accompanies the effects of ICI therapy (Fig. 4J) [2]. Additionally, CD38 expression in these naive subclusters was elevated in cancer-free survivors and decreased in untreated cancer patients further confirming the significant shift towards naive phenotypes after cancer eradication (Additional File 2: Supplementary Figure S15). Contrary to these changes, frequencies of B_4, B_7 and B_14 cells are increased in untreated LS cancer patients and are less frequent in ICI-treated patients and cancer-free survivors. Though still expressing IgD, these subclusters lost CD38 expression and started to gain CD27 expression. In particular, B_4 is the most frequent CD27^+^ B cell subcluster, mirroring a non-switched memory phenotype [32]. The cancer-specific increase in these more differentiated B cells and the decrease of naive B cells probably represent a shift towards a more activated phenotype (Fig. 4J).

### Cancer-related phenotypic alterations in peripheral immune cells reflect a response to immune-checkpoint inhibitor therapy in LS

**Fig. 5:**
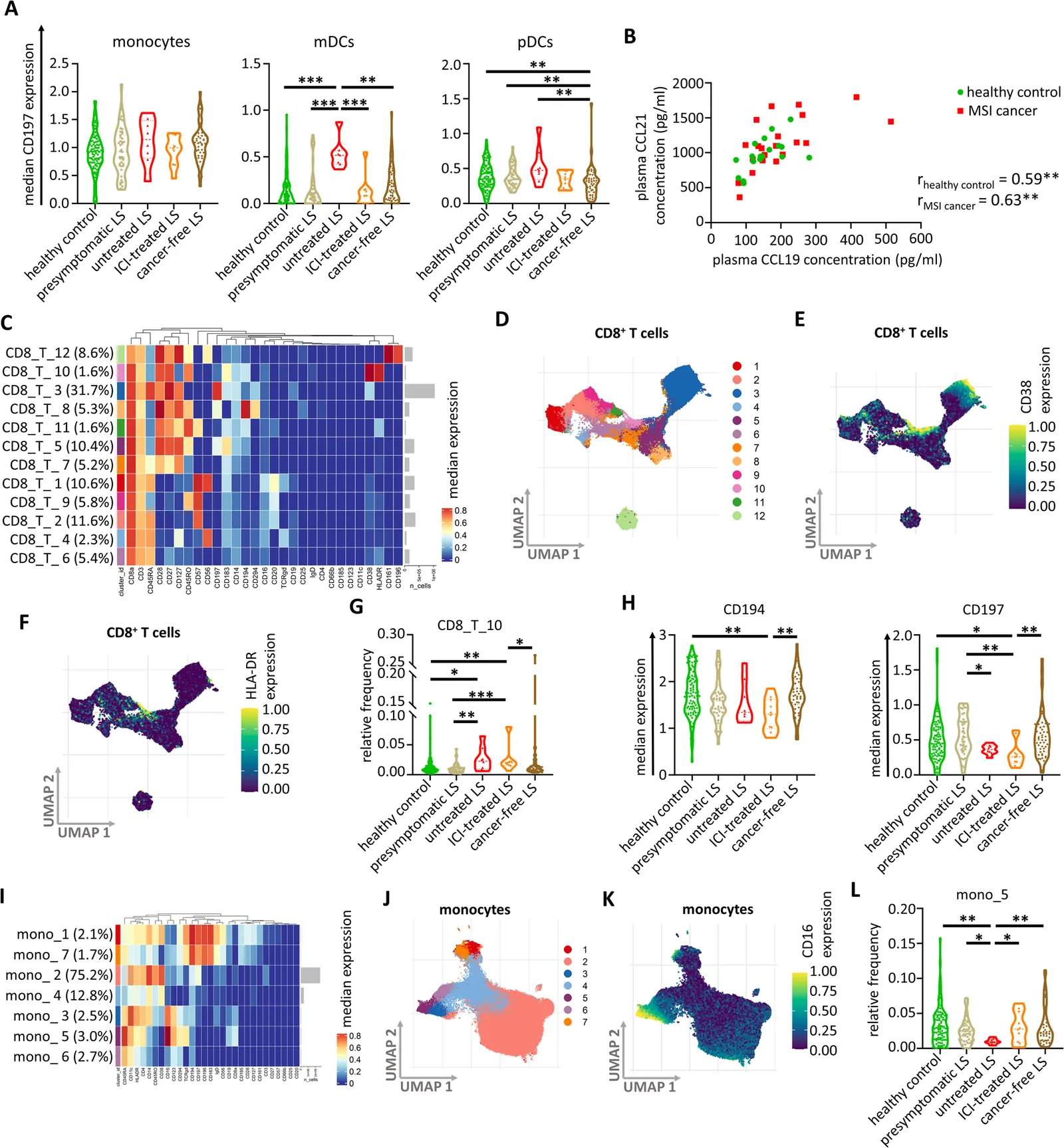
Detailed phenotypic analyses of cancer-related alterations in peripheral immune cells in Lynch syndrome. Median marker expressions of CD197 on monocytes, mDCs and pDCs in each subject (A). Extended data including age-related analysis are presented in Additional File 2: Supplementary Figure S18. Plasma CCL19 and CCL21 levels in patients with untreated microsatellite-unstable cancer and age-matched healthy controls (B). R values of Pearson’s correlation analyses are also demonstrated. Extended data including baseline characteristics of this cohort can be found in Additional File 1: Supplementary Table S10 and Additional File 2: Supplementary Figure S19. Heatmap of median marker expressions and relative frequencies of each identified peripheral CD8^+^ T cell subclusters (C). UMAP representation of peripheral CD8^+^ T cell subclusters (D). UMAP representation of peripheral CD8^+^ T cells color-coded by CD38 (E) and HLA-DR (F) expressions. Extended data for further marker expressions can be found in Additional File 2: Supplementary Figure S20. Comparison of relative frequencies of CD8_T_10 subclusters between study groups (G). Extended data including analyses of further CD8^+^ T cell subclusters with altered relative frequencies between study groups and age-related analyses are presented in Additional File 2: Supplementary Figures S21-S22. Comparison of median marker expressions of CD194, CD28 and CD197 on CD8^+^ T cell subcluster CD8_T_10 between study groups (H). Heatmap of median marker expressions and relative frequencies of each identified peripheral monocyte subclusters (I). UMAP representation of peripheral monocyte subclusters (J). UMAP representation of peripheral monocytes color-coded by CD16 expression (K). Extended data for further marker expressions can be found in Additional File 2: Supplementary Figure S23. Comparison of relative frequencies of mono_5 subclusters between study groups (L). Extended data including age-related analysis are presented in Additional File 2: Supplementary Figure S24. On violin plots, dashed lines correspond to median values, while dotted lines border interquartile ranges. Asterisks correspond to statistical significance: *p* < 0.05*; < 0.01**; < 0.001***. Abbreviations: mDCs myeloid dendritic cells, pDCs plasmacytoid dendritic cells

Next, we investigated expression differences in other immune cell populations. Within T cell subpopulations, multiple markers, whose expression correlates with naive-memory-transition (e.g. CD45RA, CD45RO) exhibited differences between study groups, but these largely correlated with the age of subjects (Additional File 2: Supplementary Figures S16-S17). No significant differences in median marker expressions were observed in NK cells. As B cells exhibited striking age-independent expression changes in various markers between study groups, we further focused on additional professional antigen-presenting cells (APCs), including monocytes, myeloid (mDCs) and plasmacytoid (pDCs) dendritic cells. CD197 was the only marker that systematically exhibited alterations in expression levels between study groups (Fig. 5A, Additional File 2: Supplementary Figure S18). Both in pDCs and mDCs, untreated cancer patients were characterized by a 1.8-2.8-fold increase in CD197 expression compared to cancer-free individuals with LS (pDC mean CD197 expression in untreated LS: 0.53 ± 0.27, in cancer-free LS: 0.30 ± 0.23, *p* = 0.005, mDC mean CD197 expression in untreated LS: 0.53 ± 0.17, in cancer-free LS: 0.19 ± 0.21, *p* = 0.002). CD197 is also known as C-C chemokine receptor type 7 (CCR7) and is upregulated following activation stimuli in professional APCs [33]. Chemokines CCL19 and CCL21 direct CCR7^+^ APCs to secondary lymphatic organs including lymph nodes [33]. Since CD197 expression was significantly elevated in 3 (B cells, mDCs and pDCs) out of 4 professional APCs in untreated cancer patients, we aimed to investigate whether this activated migratory pattern to secondary lymphatic tissues might also be characterized by elevated plasma CCL19 and CCL21 levels. To this aim we performed ELISA-based analysis on plasma samples from 21 untreated microsatellite-unstable cancer patients and 19 healthy, age-matched controls (Additional File 1: Supplementary Table S10, Additional File 2: Supplementary Figure S19). Plasma CCL19 and CCL21 levels exhibited strong correlation in both study groups and although tendentious elevations in cancer patients were observed, these alterations were not significant in this small cohort (p(CCL19) = 0.208, p(CCL21) = 0.120, Fig. 5B, Additional File 2: Supplementary Figure S19).

To analyze immune cell subsets within the lymphoid and myeloid lineages, following subclustering on CD4^+^ and CD8^+^ T cells, NK cells and monocytes we focused on those subclusters which exhibited age-independent differences in their respective relative frequencies between study groups. Among CD8^+^ T cells relative frequencies of 4 subclusters exhibited significant differences between study groups, however, only those of a smaller subcluster, CD8_T_10, characterized by high CD38 and HLA-DR expressions were unrelated to the age of participants (Fig. 5C-G, Additional File 2: Supplementary Figures S20-S22). This subcluster is expanded in both untreated and ICI-treated LS patients. Both HLA-DR and CD38 expression on CD8^+^ T cells are elevated in systemic immune activation reflecting high cytotoxic potential [34]. The robust decreases in CD194, CD28 and CD197 expressions in this subcluster in ICI-treated LS patients highlight their functionally relevant nature in immunotherapy (Fig. 5H). Within monocytes, a relatively small subcluster (subcluster mono_5) with the highest CD16 expression and low CD14 expression, resembling nonclassical monocytes was less frequent (3-fold decrease) in untreated LS patients compared to other study groups (Fig. 5I-L, Additional File 2: Supplementary Figures S23-S24). Although the frequency of this subcluster did correlate with the age of study subjects (Additional File 2: Supplementary Figure S24), this does not explain the three-fold decrease in untreated cancer patients.

### Multiparametric peripheral cytokine profiling reveals differences between individuals with LS and healthy individuals without hereditary cancer predisposition

**Fig. 6:**
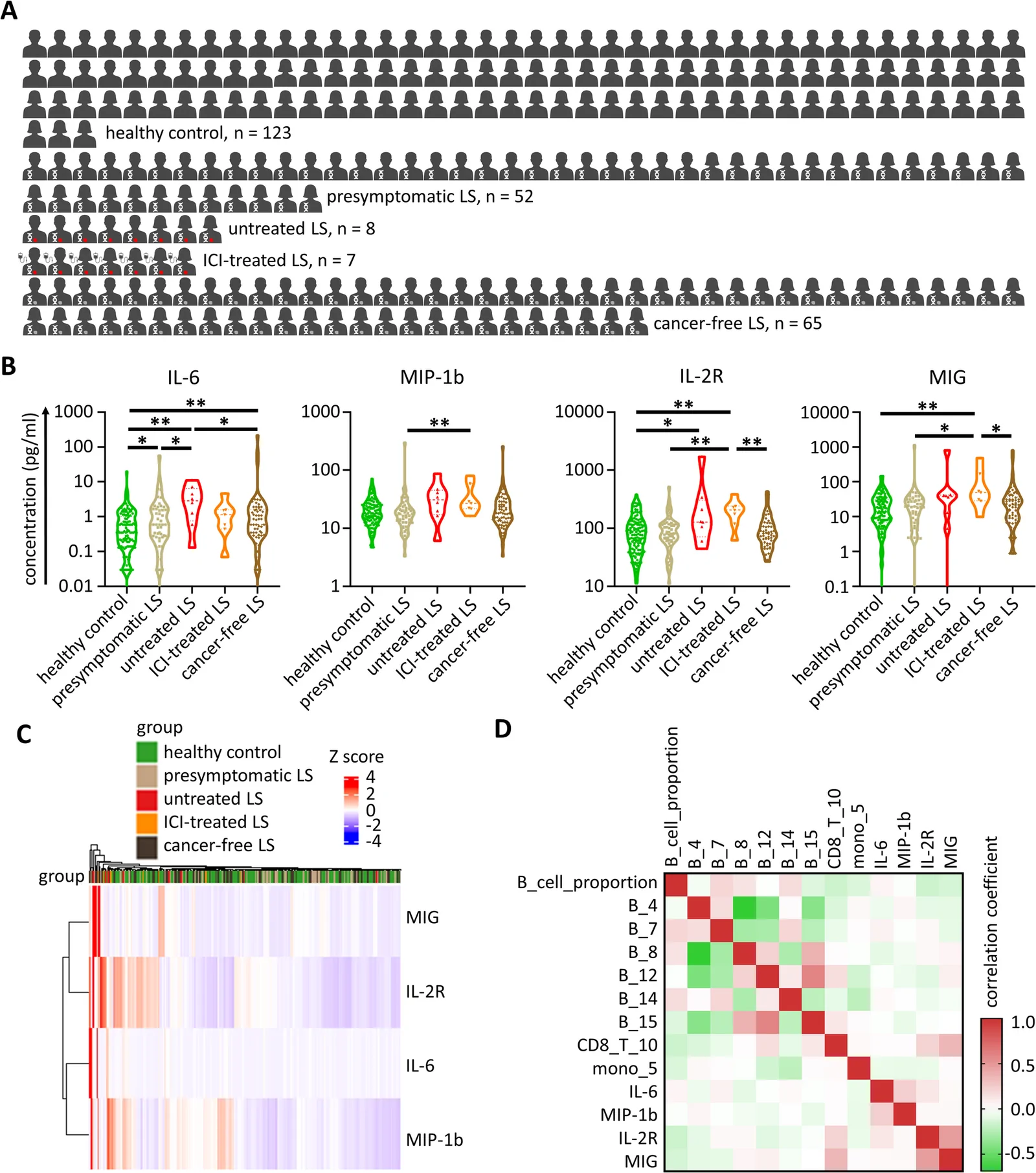
Peripheral blood cytokine profiling in LS. Graphical representation of study participants, extended data can be found in Additional File 1: Supplementary Table S11 and Additional File 2: Supplementary Figure S25 (A). Comparison of plasma cytokine and chemokine concentrations between study groups (B). Extended data for further cytokines can be found in Additional File 2: Supplementary Figure S26. Heatmap of plasma MIG, IL-2R, IL-6, MIP-1b expression levels of individuals included in the LS multiplex cytokine measurements (C). Heatmap of pairwise Pearson’s correlation coefficient values between each investigated immune cell population and plasma cytokine level (D). On violin plots, dashed lines correspond to median values, while dotted lines border interquartile ranges. Asterisks correspond to statistical significance: *p* < 0.05*; < 0.01**. Abbreviations: IL-2R interleukin-2 receptor, IL-6 interleukin-6, MIG monokine induced by gamma interferon, MIP-1b macrophage inflammatory protein 1-beta

To investigate if the robust differences in peripheral white blood cell phenotypes of LS patients are reflected in the systemic cytokine milieu, we performed plasma-based profiling of 13 cytokines/chemokines in 255 individuals, among whom 132 were diagnosed with LS (Fig. 6A, Additional File 1: Supplementary Table S11, Additional File 2: Supplementary Figure S25). 4 out of 13 cytokines exhibited altered expression between study groups (Fig. 6B, Additional File 2: Supplementary Figure S26). Levels of interleukin-2 receptor (IL-2R), macrophage inflammatory protein 1-beta (MIP-1b) and monokine induced by gamma interferon (MIG) were mainly elevated in patients with cancer. The context-dependent immunomodulatory cytokine interleukin-6 (IL-6) exhibited 1.7-fold increase in presymptomatic and cancer-free LS groups and 8.2-3.3-fold increases in untreated and ICI-treated cancer patients compared to healthy controls (median concentrations: healthy controls: 0.35 (0.14–1.01) pg/ml, vs. presymptomatic LS: 0.59 (0.26–1.67) pg/ml, *p* = 0.031; vs. untreated LS cancer patients: 2.87 (0.76–6.44) pg/ml, *p* = 0.001; vs. ICI-treated LS patients: 1.15 (0.45–1.60) pg/ml, *p* = 0.077; vs. cancer-free LS: 0.59 (0.29–1.66) pg/ml, *p* = 0.008). This signals a gradual increase in systemic inflammatory environment during carcinogenesis in LS with the first alterations already present in healthy individuals living with LS. Clustering samples based on plasma levels of these four cytokines revealed that samples from some healthy LS individuals clustered together with those of active cancer patients (Fig. 6C).

Finally, we performed a correlation analysis to investigate if relative frequencies of those cell populations which exhibited differences in their abundances between study groups correlate with levels of IL-6, MIP-1b, IL-2R and MIG measured from the same individuals. As expected, relative frequencies of several B cell subclusters correlated well with each other. Moreover, strong correlations also occurred between cytokines and cell subpopulations (Fig. 6D). In particular, the strongest such positive correlations occurred between the levels of MIG, IL-2R and the CD8^+^ T cell subcluster CD8_T_10 (r(IL-2R_CD8_T_10): 0.214, *p* = 0.005, r(MIG_CD8_T_10): 0.348 *p* = 5.63e10^− 6^). CD8_T_10 is a highly activated, cytotoxic HLA-DR^+^, CD38^+^ CD8^+^ T cell subcluster, while both soluble IL-2R and MIG are surrogate markers of CD8^+^ T cell activation [34, 35].

## Discussion

Investigating hallmarks of cancer in high-risk individuals living with HCPSs provides extraordinary opportunities to gather novel insights into carcinogenesis. It can open new research directions leading to therapeutic innovations for sporadic cancer as well (e.g. poly (ADP-ribose) polymerase inhibitors in homologous recombination-deficient or ICI therapies in dMMR cancers) [36–38]. Tumor-promoting inflammation and avoidance of immune destruction are two key hallmarks of cancer dependent on effective immunosurveillance [38]. Previous studies have confirmed tissue-level alterations of immune homeostasis in at-risk organs in healthy individuals living with HCPSs. In particular, healthy women living with gpath(*BRCA1*) exhibited elevated immune cell densities in both breast and fallopian tube [39, 40], while this phenomenon was also confirmed in colonic mucosa samples from cancer-free individuals with LS [41].

However, investigation of systemic immune phenotypes in individuals living with HCPSs is lacking. Our previous pilot study revealed increased proportions of peripheral activated CD4^+^ T cell and B cell phenotypes in healthy women living with gpath(*BRCA1*) reflective of those observed in patients with TNBC [16]. In order to more thoroughly characterize this phenotype and to investigate if systemic immune activation is present in other HCPSs we designed a comprehensive investigation of peripheral immune phenotypes in HCPSs.

Applying single-cell transcriptomics in an independent cohort, we successfully confirmed the elevated frequencies of specific activated peripheral CD4^+^ T and B cells in healthy women with gpath(*BRCA1*), which were similar to women with TNBC. Transcriptomic analyses also allowed the more granular characterization of these cell types. Based on these results, we found that two pro-inflammatory cell types, a Th17-polarized CD4^+^ T cell subpopulation and a class-switched, inflammation-adapted B cell subpopulation exhibit stronger cellular interactions, possibly mediated by the cytokine MIF [42]. Systems-level analyses of activation and differentiation within main peripheral immune subsets revealed a universally activated immune phenotype across main cell lineages highly similar between healthy women and TNBC patients living with gpath(*BRCA1*). These results all point to a systemically activated immune phenotype in gpath(*BRCA1*) women with clear implications for intervention. In particular, *BRCA1* gene therapy has been shown to reduce systemic inflammatory response and improve survival in an experimental sepsis model [43].

By investigating five study groups in LS, we aimed to model disease stages in individuals with LS. Although time-course characterization of immune phenotypes in LS would require a large-scale and long-term prospective study, the lack of available cross-sectional studies in this field makes this approach highly relevant. In contrast to HBOC, cellular phenotypes of presymptomatic carriers of LS were not altered. Cancer patients, however, exhibited clear alterations in multiple immune cell populations. Among B cells, the orchestrated shift towards a memory phenotype in cancer patients is reflected by increases in the relative frequencies of three memory and decreases in the proportions of three naive cell subsets. This shift is strikingly reversed in patients receiving ICI therapy and in cancer-free survivors. The comprehensive analysis of B cell phenotypes in these two latter study groups including the elevated transitional B cell frequencies and the normalized peripheral total B cell frequencies points to an increased efflux of naive B cells from the bone marrow reconstructing systemic immune homeostasis. While a decrease in peripheral B cell proportions in cancer patients has not been universally confirmed, earlier studies have also found a polarization towards a more activated/memory phenotype [44–46].

The dynamics of peripheral B cell populations observed in our dataset can be interpreted within the framework of established immune regulatory mechanisms. PD-1 inhibition reduces T cell exhaustion, thereby enhancing T cell–mediated interactions at the immunological synapse [47]. Effective germinal center reaction within the tumor microenvironment is also dependent on the availability of respective B cells resulting in clinically observed superior prognosis under ICI therapies [48]. In our study, patients undergoing ICI therapy exhibited an expansion of less differentiated B cell populations in the periphery, including naive-like subsets (Fig. 4I, B_8) including transitional B cells (IgD⁺CD38⁺CD20⁺, B_12, B_15) [31]. Transitional B cells directly stem from the bone marrow [31] and increases in their frequencies probably reflect an increased efflux of naive B cells from the bone marrow in reponse to ICI therapy. Clinical observations also support the key involvement of B cells both at the systemic and the tissue level following successful ICI therapies [2, 49]. On the systemic level, an increase of naive B cells were associated with better clinical responses [49], while on the tissue level Cercek et al. found that B cells start to populate cancer niches after three months of dostarlimab treatment of colorectal cancer [2].

In contrast to B cells, effective cytotoxic responses facilitated by ICI therapies are performed by terminal effector CD8^+^ T cells. An HLA-DR^+^CD38^+^CD45RO^+^ CD8^+^ T cell subpopulation (CD8_T_10) reflecting this phenotype exhibited a clearly increased relative frequency in LS patients with cancer. The phenotypic shift of this subcluster in the ICI-treated cohort further confirmed its involvement in ICI-induced cancer eradication. The striking decrease of nonclassical monocytes in untreated cancer patients was somewhat unexpected. Although a migratory pattern of nonclassical monocytes to tumor tissue has been confirmed in lymphoma, results from solid tumors are inconclusive [50, 51]. It is worth noting the striking increase of CD197/CCR7 expression in three (B cells, mDCs, pDCs) out of four professional APCs in untreated cancer patients. CCR7 expression is clearly indicative of an increased chemotactic phenotype of APCs towards secondary lymphatic tissues for germinal center reaction [33]. This result should also be interpreted with the shift to memory B cell phenotypes in the systemic immune environment of untreated LS patients as both support their orchestrated role in germinal center reaction.

Interestingly, the largest phenotypic differences were observed in B cells rather than T cells. Both LS and ICI-related research has primarily focused on antitumor T cell responses, as cytotoxic CD8^+^ T cells are the primary effectors in cellular adaptive immunity. Presymptomatic and cancer-related alterations have been systematically observed on the tissue level [41], while on the systemic level, neoantigen-specific T cells were confirmed in presymptomatic LS individuals [13]. Further studies are needed to investigate if further immune lineages might also exhibit clear phenotypic shifts in these conditions on the systemic level. One explanation might include that tissue-resident T cells can elicit effective antitumor effects under ICI therapies without a large influx of T cells from the circulation. Nevertheless, our results would support a model in which an influx of systemic B cells are needed for optimal immune response, in line with clinical observations [2, 49].

Our study design allowed the parallel investigation of the systemic cytokine milieu in individuals living with HCPSs. In contrast to universal shifts towards more differentiated cellular phenotpyes in women living with gpath(*BRCA1*), the cytokine environment in these individuals was unaltered. One explanation for this phenomenon could be that chronic low-level immune activation can only be detected by compartmentalized approaches (i.e. phenotypic alterations are exhibited in specific cellular subclusters but not in the whole leukocyte population), not reflected in total cytokine levels. Follow-up proteomic analyses would be able to specifically characterize the immune-related plasma cargo [52]. In LS, however, 4 out of the 13 investigated cytokines exhibited altered plasma concentrations between study groups, among which IL-2R and MIG were mainly elevated in ICI-treated LS patients. Earlier studies have found that these cytokines are surrogate markers for CD8^+^ T cell activation [34, 35]. Our own results also showed that the levels of these two cytokines correlated well with relative frequencies of the HLA-DR^+^CD38^+^ activated CD8^+^ T cell subcluster (CD8_T_10), while the highest levels of IL-2R and MIG in the ICI-treated group confirm the functional activation in the ICI-treated group. Our most interesting result in presymptomatic LS individuals is the elevated plasma IL-6 concentrations observed in this cohort compared to healthy controls. The elevated levels are clearly similar to those of cancer-free survivors, while they are much higher in LS patients with cancer. In the general population, a UK Biobank-based study showed that the elevation of the systemic inflammation marker C-reactive protein correlates with increased cancer risk [53]. Moreover, the well-known nonsteroidal anti-inflammatory drug aspirin has been confirmed to decrease cancer risk in individuals with LS [54]. Considering the context-dependent consequences of IL-6 with regard to cancer (i.e. low-level, intermittent increase supports antitumor immunity, while tonic, high-level elevations are tumorigenic) [55, 56], further analyses would be needed to characterize the biological consequences of these observed alterations especially with regard to aspirin chemoprevention.

It is important to note the limitations of this study. Reflecting the monocentric, clinical setting of the studied cohorts, a lower number of untreated cancer patients were included as compared to healthy carriers living with HCPSs. Results from monocentric, cross-sectional studies are needed to be validated in a prospective, multicentric manner on independent cohorts. In addition to age, confounding factors might include sex, affected mismatch repair gene, and lifestyle factors. Although we have found no significant differences in the distribution of sex, affected mismatch repair gene and previous cancer histories between respective LS cohorts, we cannot rule out their role in affecting our results. Additionally, the investigation of peripheral levels of 13 cytokines cannot recapitulate the overall systemic cytokine environment.

## Conclusions

In summary, we performed a comprehensive systemic immunophenotyping study in 227 individuals with HCPS and 164 healthy controls. In women with gpath(*BRCA1*), we described elevated frequencies of a Th17-polarized CD4^+^ T cell and a proinflammatory B cell subpopulation in healthy women and in TNBC patients, while this was accompanied by more differentiated phenotypes in additional immune lineages, especially CD8^+^ T cells. In LS, a cancer-related shift towards a memory phenotype in B cells was found, which was reversed in ICI-treated patients and cancer-free survivors. Importantly, elevated plasma IL-6 levels were detected in presymptomatic individuals with LS, which were further elevated in LS patients with cancer. The systemic immunological changes observed in presymptomatic carriers of HCPSs point to functionally relevant immunosurveillance targets which could be leveraged in personalized and targeted risk-reducing strategies. The alterations of peripheral cellular and humoral immune phenotypes might contribute to DNA-based early cancer detection strategies and elevate their sensitivities and specificities. Moreover, if prospectively validated, the increases of naive B cell proportions in response to ICI therapies might serve as surrogate markers for immunotherapy response.

## Supplementary Information

Below is the link to the electronic supplementary material.


Supplementary Material 1: Supplementary Table S1–S11



Supplementary Material 2: Supplementary Figure S1–S26


## Data Availability

All data generated or analysed during this study are included in this published article, have been deposited [57] at Zenodo (https://doi.org/10.5281/zenodo.18268936) and will be made public upon publication.

## Declarations

### Statement of significance

By the comprehensive systemic immune profiling of 391 individuals, among whom 227 are living with hereditary cancer predisposition syndromes, we report specific immune activation phenotypes even in healthy individuals living with high genetic risk to cancer, which provides new targets for personalized cancer interception.

## Abbreviations

APC: antigen–presenting cell
CCL: chemokine ligand pathway
CCL11: C–C motif chemokine 11
CCR7: C–C chemokine receptor type 7
CD70: cluster of differentiation 70
CRC: colorectal cancer
CypA: cyclophilin A pathway
dMMR: mismatch repair–deficient EC–endometrial cancer
EM: effector memory CD8^+^ T cells
FBS: fetal bovine serum
gpath: germline pathogenic variant
HBOC: hereditary breast and ovarian cancer syndrome
HCPS: hereditary cancer predisposition syndrome
ICI: immune checkpoint inhibitor
IFNg: interferon gamma
IL: 1b–interleukin–1 beta
IL: 1RA–interleukin–1 receptor antagonist
IL: 2–interleukin–2
IL: 2R–interleukin–2 receptor
IL: 6–interleukin–6
IL: 12–interleukin–12
IL16: interleukin 16 pathway
IP: 10–interferon gamma–induced protein 10
LS: Lynch syndrome
MAIT: mucosal–associated invariant T cells
MCP: 1–monocyte chemoattractant protein 1
MCSB: Maxpar cell staining buffer
mDC: myeloid dendritic cell
MIF: macrophage migration inhibitory factor
MIG: monokine induced by gamma interferon
MIP: 1a–macrophage inflammatory protein 1–alpha
MIP: 1b–macrophage inflammatory protein 1–beta
NK: natural killer
PAS: pathway activity score
PBMC: peripheral blood mononuclear cell
pDC: plasmacytoid dendritic cell
RT: room temperature
TE: terminal effector CD8^+^ T cells
TGFb: transforming growth factor beta–pathway
TNBC: triple–negative breast cancer
TNFa: tumor necrosis factor alpha
t: SNE–t–distributed stochastic neighbor embedding
UMAP: uniform manifold approximation and projection VISFN–visfatin–pathway
