# Supplementary material for: Systemic immune activation in hereditary cancer predisposition syndromes: a cross-sectional study
Source: BMC Med. 2026 Aug 25;24:467. doi: 10.1186/s12916-026-05188-x (PMC13523197; doi:10.1186/s12916-026-05188-x)
Supplement: Supplementary file 1 [file 12916_2026_5188_MOESM2_ESM.docx]

**Systemic immune activation in hereditary cancer predisposition syndromes: a cross-sectional study**

István Kelemen, Klaudia Horti-Oravecz, Anikó Bozsik, Tímea Pócza, János Papp, István Likó, Patrícia Neuperger, Fanni Balogh, Ágnes Kemény, Petra Nagy, Viktória Vereczki, Szonja Polett Pósa, Lőrinc Sándor Pongor, Dorottya Kövesdi, Henriett Butz, Attila Patócs, Gábor János Szebeni, Vince Kornél Grolmusz

**Additional File 2: Supplementary Figures**

**Supplementary Figure S1**

**Supplementary Figure S1 – Comparison of the age of participants and analyzed cells/sample between study groups in the single-cell transcriptomics study.**

**Supplementary Figure S2A**

CD4+ T cell sublcusters

**Supplementary Figure S2 – Heatmap based on gene expression of the top 10 marker genes of CD4^+^ T cell (A) and B cell (B) subclusters.**

**Supplementary Figure S2B**

B cell sublcusters

**Supplementary Figure S3**

**Supplementary Figure S3 – Comparison of the age of participants between study groups in the MIF ELISA measurements.** On violin plots, dashed lines correspond to median values, while dotted lines border interquartile ranges

**Supplementary Figure S4**

**Supplementary Figure S4 – Comparison of differentiation scores of CD4^+^ T cells (A), B cells (B), NK cells (C) and monocytes (D) between study groups.** On violin plots, dashed lines correspond to median values, while dotted lines border interquartile ranges. Asterisks correspond to statistical significance: p < 0.05*; < 0.01**; < 0.001***. Extended data for Figure 3I.

**Supplementary Figure S5**

**Supplementary Figure S5 – Comparison of the age of healthy controls, gpath(*BRCA1*) healthy and gpath(*BRCA1*) TNBC women included in the multiplex cytokine measurements.** On violin plots, dashed lines correspond to median values, while dotted lines border interquartile ranges.

**Supplementary Figure S6**

**Supplementary Figure S6 – Heatmap of plasma cytokine and chemokine expression levels of healthy control, gpath(*BRCA1*) healthy and gpath(*BRCA1*) TNBC women included in the multiplex cytokine measurements.** Abbreviations: CCL11 – C-C motif chemokine 11, IL-1b – interleukin-1 beta, IL-1RA – interleukin-1 receptor antagonist, IL-2 – interleukin-2, IL-2R – interleukin-2 receptor, IL-6 – interleukin-6, IL-12 – interleukin-12, IP-10 – interferon gamma-induced protein 10, MCP-1 – monocyte chemoattractant protein 1, MIG – monokine induced by gamma interferon, MIP-1a – macrophage inflammatory protein 1-alpha, MIP-1b – macrophage inflammatory protein 1-beta, TNFa – tumor necrosis factor alpha.

**Supplementary Figure S7**

**Supplementary Figure S7 – Comparison of plasma cytokine and chemokine concentrations of healthy control, gpath(*BRCA1*) healthy and gpath(*BRCA1*) TNBC women included in the multiplex cytokine measurements.** Abbreviations: CCL11 – C-C motif chemokine 11, IL-1b – interleukin-1 beta, IL-1RA – interleukin-1 receptor antagonist, IL-2 – interleukin-2, IL-2R – interleukin-2 receptor, IL-6 – interleukin-6, IL-12 – interleukin-12, IP-10 – interferon gamma-induced protein 10, MCP-1 – monocyte chemoattractant protein 1, MIG – monokine induced by gamma interferon, MIP-1a – macrophage inflammatory protein 1-alpha, MIP-1b – macrophage inflammatory protein 1-beta, TNFa – tumor necrosis factor alpha. On violin plots, dashed lines correspond to median values, while dotted lines border interquartile ranges.

**Supplementary Figure S8**

**Supplementary Figure S8 – Comparison of the age of individuals included in the mass cytometry phenotyping measurements.** Comparison of subjects’ age between healthy control (n = 91) and LS (n = 108) cohorts (A). Comparison of subjects’ age between healthy control (n = 91) and LS subgroups: presymptomatic (n = 43), patients with untreated cancer (n = 8), patients with cancer undergoing pembrolizumab-treatment (ICI-treated, n = 10) and cancer-free survivors (n = 47, B). On violin plots, dashed lines correspond to median values, while dotted lines border interquartile ranges. Asterisks correspond to statistical significance: p < 0.05*; < 0.01**; < 0.001***. Further characteristics can be found in Additional File 1: Supplementary Table S8.

**Supplementary Figure S9**

**Supplementary Figure S9 – t-SNE and UMAP representation of peripheral immune cells based on the mass cytometry profiling.** Extended data to Figure 4B.

**Supplementary Figure S10**

**Supplementary Figure S10 – UMAP representation of peripheral immune cells based on the mass cytometry profiling color-coded by expressions of the investigated 29 cell surface markers.** Extended data to Figure 4B.

**Supplementary Figure S11**

**Supplementary Figure S11 – Correlation of peripheral B cell proportions with the age of the subjects in various study groups** Results from Pearson’s correlation analyses (r, r^2^, p-values) are presented for each study group. Extended data for Figure 4D.

**Supplementary Figure S12**

**Supplementary Figure S12 – Correlation of median marker expressions on peripheral B cells with the age of the subjects in various study groups** Results from Pearson’s correlation analyses (r, r^2^, p-values) are presented for each study group. Extended data for Figure 4E.

**Supplementary Figure S13**

**Supplementary Figure S13 – UMAP representation of peripheral B cells color-coded by expressions of the investigated 29 cell surface markers.** Extended data to Figure 4G.

**Supplementary Figure S14**

**Supplementary Figure S14 – Correlation of relative frequencies of peripheral B cell subclusters B_4, B_7, B_8, B_12, B_14 and B_15 with the age of the subjects in various study groups** Results from Pearson’s correlation analyses (r, r^2^, p-values) are presented for each study group. Extended data for Figure 4I.

**Supplementary Figure S15**

**Supplementary Figure S15 – Comparison of median marker expressions of CD38 on B cell subclusters B_8 and B_12 between study groups** On violin plots, dashed lines correspond to median values, while dotted lines border interquartile ranges. Asterisks correspond to statistical significance: p < 0.05*; < 0.01**.

**Supplementary Figure S16**

**Supplementary Figure S16 – Comparison of median marker expressions of significantly differently expressed markers by study groups on T cells** On violin plots, dashed lines correspond to median values, while dotted lines border interquartile ranges. Asterisks correspond to statistical significance: p < 0.05*; < 0.01**; < 0.001***. Abbreviations: DNT cells – double negative T cells, TCRgd cells – γδT cells. Extended data including age-related analyses are presented in Additional File 2: Supplementary Figure S17.

**Supplementary Figure S17**

**Supplementary Figure S17 – Correlation of median marker expressions of markers from Supplementary Figure S16 with the age of the subjects in various study groups** Results from Pearson’s correlation analyses (r, r^2^, p-values) are presented for each study group. Extended data for Additional File 2: Supplementary Figure S16.

**Supplementary Figure S18**

**Supplementary Figure S18 – Correlation of median CD197 expressions with the age of the subjects in monocytes, mDCS and pDCs** Results from Pearson’s correlation analyses (r, r^2^, p-values) are presented for each study group. Abbreviations: mDCs – myeloid dendritic cells, pDCs – plasmacytoid dendritic cells. Extended data for Figure 5A.

**Supplementary Figure S19**

**Supplementary Figure S19 – Results from plasma CCL19 and CCL21 expression analyses in patients with microsatellite-unstable cancer** Comparison of age of individuals included in the ELISA measurements (A). Plasma CCL19 concentrations in patients with untreated microsatellite-unstable cancer and healthy controls (B). Plasma CCL21 concentrations in patients with untreated microsatellite-unstable cancer and healthy controls (C). On violin plots, dashed lines correspond to median values, while dotted lines border interquartile ranges. Further characteristics can be found in Additional File 1: Supplementary Table S10.

**Supplementary Figure S20**

**Supplementary Figure S20 – UMAP representation of peripheral CD8^+^ T cells color-coded by expressions of the investigated 29 cell surface markers.** Extended data to Figures 5E-F.

**Supplementary Figure S21**

**Supplementary Figure S21 – Relative frequencies of CD8^+^ T cell subclusters with significant changes in relative frequencies between study groups** On violin plots, dashed lines correspond to median values, while dotted lines border interquartile ranges. Asterisks correspond to statistical significance: p < 0.01**; < 0.001***. Extended data for Figure 5G.

**Supplementary Figure S22**

**Supplementary Figure S22 – Correlation of relative frequencies of peripheral CD8^+^ T cell subclusters SC3, SC8, SC10 and SC11 with the age of the subjects in various study groups** Results from Pearson’s correlation analyses (r, r^2^, p-values) are presented for each study group. Extended data for Figure 5G.

**Supplementary Figure S23**

**Supplementary Figure S23 – UMAP representation of peripheral monocytes color-coded by expressions of the investigated 29 cell surface markers.** Extended data to Figure 5K.

**Supplementary Figure S24**

**Supplementary Figure S24 – Correlation of relative frequencies of peripheral monocyte subcluster SC5 with the age of the subjects in various study groups** Results from Pearson’s correlation analyses (r, r^2^, p-values) are presented for each study group. Extended data for Figure 5L.

**Supplementary Figure S25**

**Supplementary Figure S25 – Comparison of the age of individuals included in the LS peripheral cytokine profiling measurements.** Comparison of subjects’ age between healthy control (n = 123) and LS (n = 132) cohorts (A). Comparison of subjects’ age between healthy control (n = 123) and LS subgroups: presymptomatic LS (n = 52), patients with untreated cancer (n = 8), patients with cancer undergoing pembrolizumab-treatment (ICI-treated, n = 7) and cancer-free individuals living with LS (n = 65, B). On violin plots, dashed lines correspond to median values, while dotted lines border interquartile ranges. Asterisks correspond to statistical significance: p < 0.05*; < 0.01**; < 0.001***. Further characteristics can be found in Additional File 1: Supplementary Table S11.

**Supplementary Figure S26**

**Supplementary Figure S26 – Comparison of plasma cytokine and chemokine concentrations of LS study groups** Extended data for Figure 6B. Abbreviations: CCL11 – C-C motif chemokine 11, IL-1b – interleukin-1 beta, IL-1RA – interleukin-1 receptor antagonist, IL-2 – interleukin-2, IL-12 – interleukin-12, IP-10 – interferon gamma-induced protein 10, MCP-1 – monocyte chemoattractant protein 1, MIP-1a – macrophage inflammatory protein 1-alpha, TNFa – tumor necrosis factor alpha. On violin plots, dashed lines correspond to median values, while dotted lines border interquartile ranges.

**Legends for Additional File 1**

**Supplementary Table S1 – Baseline characteristics of participants in the single-cell transcriptomics study.** Note: Variants correspond to the MANE Select transcript of *BRCA1* (NM_007294.4).

**Supplementary Table S2** **– Significantly differently expressed genes in CD4_T_8 subcluster compared to other CD4^+^ T cells** Significantly differently expressed genes (p_adj < 0.05) with |avglog2FC| > 1 are listed.

**Supplementary Table S3** **– Significantly differently expressed genes in B_1 subcluster compared to other B cells** Significantly differently expressed genes (p_adj < 0.05) with |avglog2FC| > 1 are listed.

**Supplementary Table S4 – Baseline characteristics of participants in the MIF ELISA measurements.** Note: Variants correspond to the MANE Select transcript of *BRCA1* (NM_007294.4). Note: Healthy control individuals overlap between this dataset and the dataset described in Additional File 1: Supplementary Tables S7, S8, S9 and S10, while gpath(*BRCA1*) women overlap between this dataset and the dataset described in Additional File 1: Supplementary Table S7.

**Supplementary Table S5 – Differently expressed genes within main peripheral immune populations in pairwise comparisons between healthy_control, gpath(*BRCA1*)_healthy, gpath(*BRCA1*)_TNBC study groups** Significantly differently expressed genes (p_adj < 0.05) are listed.

**Supplementary Table S6 – Immune-related pathways with significantly altered pathway activity scores as detected by AUCell between** **healthy_control, gpath(*BRCA1*)_healthy, gpath(*BRCA1*)_TNBC study groups in main immune lineages.** Pathways with significantly altered pathway activity scores (p_adj < 0.05) present in at least 30% of respective cell type/study group are listed.

**Supplementary Table S7 – Baseline characteristics of healthy controls, gpath(*BRCA1*) healthy and gpath(*BRCA1*) TNBC women included in the multiplex cytokine measurements.** Note: Variants correspond to the MANE Select transcript of *BRCA1* (NM_007294.4). Note: healthy control individuals overlap between this dataset and the dataset described in Additional File 1: Supplementary Tables S4, S8, S9 and S10, while gpath(*BRCA1*) women overlap between this dataset and the dataset described in Additional File 1: Supplementary Table S4.

**Supplementary Table S8 – Baseline characteristics of individuals included in the mass cytometry measurements.** Note: Variants correspond to the MANE Select transcript of each gene (*MLH1*: NM_000249.4; *MSH2*: NM_000251.3; *MSH6*: NM_000179.3; *PMS2*: NM_000535.7; *EPCAM*: NM_002354.3). Abbreviations: F – female, gpath – germline pathogenic variant, M – male. Note: healthy control individuals overlap between this dataset and the the dataset described in Additional File 1: Supplementary Tables S4, S7, S9 and S10, while individuals with LS overlap between this dataset and the dataset described in Additional File 1: Supplementary Tables S9 and S10.

**Supplementary Table S9 – Comparison of the distribution of sex, affected gene carrying the LS-associated gpath variant and the most recent cancer type between study groups.** Contingency tables were subjected to chi2 analyses with the resultant p-values present in each table. Abbreviations: CRC – colorectal cancer, EC – endometrial cancer, OC – ovarian cancer.

**Supplementary Table S10 – Baseline characteristics of individuals included in the CCL19 and CCL21 ELISA measurements.** Note: Variants correspond to the MANE Select transcript of each gene (*MLH1*: NM_000249.4; *MSH2*: NM_000251.3; *MSH6*: NM_000179.3; *PMS2*: NM_000535.7; *EPCAM*: NM_002354.3). Abbreviations: F – female, gpath – germline pathogenic variant, M – male. Note: healthy control individuals overlap between this dataset and the dataset described in Additional File 1: Supplementary Tables S4, S7, S8 and S10, while individuals with LS overlap between this dataset and the dataset described in Additional File 1: Supplementary Tables S8 and S10.

**Supplementary Table S11 – Baseline characteristics of individuals included in the Lynch syndrome peripheral multiplex cytokine profiling.** Note: Variants correspond to the MANE Select transcript of each gene (*MLH1*: NM_000249.4; *MSH2*: NM_000251.3; *MSH6*: NM_000179.3; *PMS2*: NM_000535.7; *EPCAM*: NM_002354.3). Abbreviations: F – female, gpath – germline pathogenic variant, M – male. Note: healthy control individuals overlap between this dataset and the dataset described in Additional File 1: Supplementary Tables S4, S7, S8 and S9, while individuals with LS overlap between this dataset and the dataset described in Additional File 1: Supplementary Tables S8 and S9.
